# A multilingual and multimodal approach to literacy teaching and learning in urban education: a collaborative inquiry project in an inner city elementary school

**DOI:** 10.3389/fpsyg.2014.00533

**Published:** 2014-06-18

**Authors:** Burcu Yaman Ntelioglou, Jennifer Fannin, Mike Montanera, Jim Cummins

**Affiliations:** ^1^Department of Curriculum, Teaching and Learning, Ontario Institute for Studies in EducationToronto, ON, Canada; ^2^Toronto District School BoardToronto, ON, Canada

**Keywords:** urban education, multilingualism, multiliteracies, multimodality, collaborative inquiry, identity texts

## Abstract

This paper presents findings from a collaborative inquiry project that explored teaching approaches that highlight the significance of multilingualism, multimodality, and multiliteracies in classrooms with high numbers of English language learners (ELLs). The research took place in an inner city elementary school with a large population of recently arrived and Canadian-born linguistically and culturally diverse students from Gambian, Indian, Mexican, Sri Lankan, Tibetan and Vietnamese backgrounds, as well as a recent wave of Roma students from Hungary. A high number of these students were from families with low-SES. The collaboration between two Grade 3 teachers and university-based researchers sought to create instructional approaches that would support students’ academic engagement and literacy learning. In this paper, we described one of the projects that took place in this class, exploring how a descriptive writing unit could be implemented in a way that connected with students’ lives and enabled them to use their home languages, through the creation of multiple texts, using creative writing, digital technologies, and drama pedagogy. This kind of multilingual and multimodal classroom practice changed the classroom dynamics and allowed the students access to identity positions of expertise, increasing their literacy investment, literacy engagement and learning.

## INTRODUCTION: STUDENTS’ HOME LANGUAGES, THE MISSING CONVERSATION EVEN IN PRO-SOCIAL JUSTICE URBAN EDUCATIONAL CONTEXTS

The 2011 Canadian census revealed that more than 200 languages were reported as immigrant home languages and 9 in 10 Canadians who speak a home language other than English or French live in cities, particularly Toronto, Montreal, Vancouver, and Calgary (Statistics Canada, 2013). This increase in linguistic diversity reflects the fact that over a period of 20 years, annual immigration to Canada has remained steady at about 250,000 per annum. Thus, linguistic diversity is becoming the norm in urban school systems across Canada. This increase in diversity highlights the obvious fact that “literacy” cannot be viewed as synonymous with English (or French) literacy. Outside of school, students and communities are engaged with multiple forms of literacies (Gee, 1996; Street, 2003), involving different languages.

Many educational researchers have addressed the need to respond to this demographic shift in the linguistic composition of Canadian classrooms (Cummins, 2000; Lotherington, 2011; Naqvi et al., 2012a). Within the prevailing educational practices of urban schools, it is clear that English language learners (ELLs) face serious challenges in achieving high literacy levels and literacy engagement (Collier, 1992, 1995a,b; August and Hakuta, 1998; Cummins, 2000), and also face the risk of losing their home languages (Wong-Fillmore, 1991; Portes and Hao, 1998; Baker, 2001; Oller and Eilers, 2002; Baca and Cervantes, 2004; Bialystok et al., 2004; Cummins, 2005). As early as 1988, Mary Ashworth called attention to the fact that, even with the multiculturalism that was being promoted in Canadian educational systems at the time, bilingual children were becoming less than they were, not more than they were – a contradiction to the purpose of education, which should exist to increase, rather than decrease, students’ potential. Empirical research in applied linguistics and language education on the use of more than one language as a medium of instruction in schools has been carried out since the 1920s. There is considerable consensus in these studies that development of literacy in two or more languages provides linguistic, cognitive, and social advantages for bilingual/multilingual students (Hornberger, 1990, 2003; Cummins, 2001; García et al., 2007; Dagenais et al., 2008; Cummins and Early, 2011; Naqvi et al., 2012a,b). As García (2009, p. 157) has argued, schools need “to recognize the multiple language practices that heterogeneous populations increasingly bring and which integrated schooling, more than any other context, has the potential to liberate.”

Unfortunately, Canadian schools have been slow to recognize the multiple language practices of their students and communities. Even in school systems that have endorsed social justice as a defining attribute of their educational philosophies (such as the Toronto District School Board TDSB), there has been little conversation about the implications of linguistic diversity for educational practice. The topic is largely absent from principals’ courses and from initial teacher education courses. Prominent books on school leadership and the management of educational change (e.g., Fullan, 2001) ignore the issue. In other words, until recently, home languages other than English or French have been viewed as largely irrelevant to children’s schooling. At best, they are treated with benign neglect and ignored; at worst, some educators still consider them an obstacle to the acquisition of English or French and discourage their use in school and at home. An example of this latter orientation is the November 2011 decision of the Commission Scolaire de Montréal (CSDM), where 47% of students speak a home language other than French or English, to mandate that all students use only French throughout the school.

The absence of serious policy consideration of issues related to linguistic diversity at all levels of the educational system has resulted in the “normalization” of certain assumptions and practices in Canadian schools serving ELL:

• Provision of instructional support for ELL students is the job of the ESL teacher;
• “Literacy” refers only to English (or French) literacy;
• The cultural knowledge and home language proficiency that ELL students bring to school have little instructional relevance;
• Culturally and linguistically diverse parents, whose English may be quite limited, do not have the language skills to contribute to their children’s literacy development.

In recent years, these normalized assumptions have been challenged by Canadian educators and researchers who have engaged in collaborative projects to articulate a very different set of pedagogical assumptions in regard to the multilingual realities of urban schools. These projects have attempted to build on and extend students’ multilingual competencies within both “mainstream” and ESL classrooms. Cummins et al. (2006) articulated the following pedagogical claims on the basis of their collaborative work:

• ELL students’ cultural knowledge and language abilities are important resources in enabling academic engagement across the curriculum;
• ELL students will engage academically to the extent that instruction affirms their identities and enables them to invest their identities in learning;
• Culturally and linguistically diverse parents represent a significant source of support for students’ literacy development in both English and the home language when literacy instructional practices in the school encourage home-school collaboration.

In recent years, there has been concentrated attention by researchers across Canada on the urban classroom reality of multilingualism. These researchers come from different geographical and theoretical places, but their findings converge on the conclusion that, with little funding but a change of outlook, mainstream classroom teachers can implement multilingual, multiliteracies pedagogies with positive results for their students.

The following projects are among those that have attempted to change the ways in which Canadian schools respond to the multi-lingual realities of their students and communities (expanded from the list provided in Cummins and Persad, 2014):

• The *ÉLODiL* project (Éveil au Langage et Ouverture à la Diversité Linguistique – Awakening to Language and Opening up to Linguistic Diversity^1^) has developed a wide variety of classroom activities to promote students’ awareness of language and appreciation of linguistic diversity. This project has been undertaken both in Montreal (Dr. Françoise Armand, Université de Montréal) and Vancouver (Dr. Diane Dagenais, Simon Fraser University; Dagenais et al., 2008; Armand and Dagenais, 2012).
• The *Dual Language Showcase*^2^ was created by educators at Thornwood Public School in the Peel District School Board near Toronto to showcase the dual language writing accomplishments of elementary school students (Chow and Cummins, 2003; Schecter and Cummins, 2003).
• The *Multiliteracies* project involved a series of collaborations between educators and university researchers in the Vancouver and Toronto areas to explore the pedagogical possibilities that emerge when conceptions of literacy within schools are broadened to take account of multilingualism, multiliteracies, and multimodalities^3^ (Early and Yeung, 2009; Cummins and Early, 2011).
• The *Multiliteracies Pedagogy* project initiated in 2003 by Dr. Heather Lotherington of York University in Toronto involved a range of collaborations between educators in Joyce Public School and researchers at York University to explore how the concept of plurilingualism could be translated into pedagogical design. The professional learning community at Joyce P. S. worked with students to rewrite traditional stories from a critical perspective using multimodal and multilingual forms of representation (Lotherington, 2011, 2013; Lotherington and Sinitskaya Ronda, 2012; Lotherington et al., 2013).
• *Linguistically Appropriate Practice (LAP)* is an approach to working with preschool and primary grade children from immigrant backgrounds, aimed at enabling children to realize their bilingual potential. Developed by Dr. Roma Chumak-Horbatsch (2012) at Ryerson University in Toronto, LAP consists of both an educational philosophy and a set of concrete instructional activities that help teachers transform their classrooms from monolingual into multilingual environments where students’ languages are acknowledged and come to life.
• *The Dual Language Reading Project* was initiated by Dr. Rahat Naqvi of the University of Calgary and colleagues in the Calgary Board of Education. It documented the linguistic and metalinguistic benefits that students experienced as a result of teachers and community members reading dual language books to students both in linguistically diverse schools and in the Calgary Board of Education’s Spanish-English bilingual program^4^ (Naqvi et al., 2012a,b).
• The *Family Treasures and Grandma’s Soup* dual language book project was initiated by Dr. Hetty Roessingh at the University of Calgary in collaboration with the Almadina Language Charter Academy, a public charter school focused on providing comprehensive language support to students learning English as an additional language. In the project, Kindergarten and Grade 1 students created dual language books as a means of enhancing their early literacy progress^5^ (Roessingh, 2011).
• Dr. Shelly Taylor at Western University, London, Ontario, conducted a dual language book project designed to produce positive identity texts to counter damaging representations of Aboriginal communities. “The participant-authors were Aboriginal parents who wrote books intended for their preschool-aged children in their ancestral language and English” (Taylor, 2011, p. 289).
• The *ScribJab* website and iPad application^6^ were created by Simon Fraser University researchers Dr. Diane Dagenais and Dr. Kelleen Toohey to enable students to read and create digital stories (text, illustrations and audio recordings) in multiple languages (English, French and other non-official languages). The website notes that “*ScribJab* creates a space for children to communicate about their stories, and come to an enhanced appreciation of their own multilingual resources.”

These projects document the possibilities of what we have called *teaching through a multilingual lens* (Cummins and Persad, 2014). They represent “bottom-up” school-based language policy initiatives in which educators challenge the restrictive normalized assumptions with respect to linguistic diversity that still predominate in schools across Canada. The collaborative project which we describe below is rooted in similar pedagogical and social philosophies; simply stated, our starting point is that instruction in multilingual and multicultural schools will be effective to the extent that it challenges societal power structures that marginalize students’ cultural and linguistic capital.

## CONTEXT AND METHODOLOGY

### METHODOLOGY AND DATA COLLECTION METHODS

The project was initiated by Jennifer Fannin and Mike Montanera, who co-taught Grades 2/3 students in this inner-city school. They questioned how, as teachers, they could build on their students’ *funds of knowledge* (Gonzalez, 1995; González et al., 2005) and promote students’ academic engagement, literacy investment, and literacy learning. They contacted Jim Cummins and Burcu Yaman Ntelioglou, university-based researchers, in October 2012 to explore possibilities for collaboration. This paper presents the findings from the resulting collaborative inquiry project. Methodologically, we decided that this project would be a *Collective Pedagogical Inquiry*. The goal of a collective pedagogical inquiry framework is for the teachers/school-based researchers and university-based educators/researchers to work collaboratively and examine the organizational and pedagogical choices that are being made in a specific context, explore possible alternatives, and mobilize the research evidence and their own pedagogical experiences both to articulate school-based language policies and collectively implement instructional and organizational changes that respond to the challenges and opportunities represented by the students and communities.

Collective Pedagogical Inquiry methodology, like Practitioner Research/Action Research (Crookes, 1993; Cochran-Smith and Lytle, 2009; Simon et al., 2012) is situated in teacher practice with the aim of researching the pedagogical questions identified by the teachers. In addition to the important aspect of the research questions coming directly from the teachers’ pedagogies and the teaching and learning in their specific classrooms, another very important aspect is the collaborative and participatory approach in Collective Pedagogical Inquiry methodology. The teachers and the researchers work collaboratively from the planning of classroom work to data analysis. Challenging the teacher/researcher dichotomy, the teachers and the researchers become co-teachers and co-researchers in all aspects of the process. The data sources for this project included observation field-notes, videotaped classroom practice, and multimodal artifacts created in the classroom (e.g., digital texts, drama performances, student writings), as well as formal and informal interviews with the teachers and the students, and individual and focus group interviews with the parents.

### CONTEXT AND PARTICIPANTS

Jennifer and Mike were teachers in a Grades K-8 school with about 550 students, 76% of whom spoke a language other than English at home. The two teachers described the school and community context as follows:

Our school is an inner city school with each class composed of around 50% Hungarian Roma students. These students are here claiming refugee status and their situation has been very tenuous. The rest of our student population comprises a high number of ELLs from different backgrounds such as Tibetan, Indian, Sri Lankan, Vietnamese, Gambian and several others. (Email communication, October, 2012)

As the teachers describe in this first email communication, the research took place in an inner city elementary school with a large population of recently arrived and Canadian-born linguistically and culturally diverse students from countries such as Gambia, India, Mexico, Sri Lanka, Tibet, and Vietnam. A high number of these students were from low-SES backgrounds and some of the families lived in the subsidized “community housing” buildings in the school’s neighborhood. The student body also included a recent wave of Roma students. Some of these students, at the time of the project, were experiencing significant language, literacy and social challenges. These challenges were compounded by the fact that these Roma students came from a social group that has been subjected to racism in their home countries and whose status, both social and legal, within Canada is marginalized and uncertain. In fact, over the course of the academic year, many of the Roma students and their families had been deported back to Hungary. Most of these students’ lack of experience or negative experience with schooling, and their uncertainty of not knowing if their families’ refugee claims would be accepted or not, all influenced both the classroom environment and students’ investment and academic engagement. Within this same email, the teachers explained that their primary goals, therefore, were to spark the students’ interest in reading and to change their attitudes toward literacy:

We are interested in creating identity texts with our students in order to increase their interest in reading and improve their attitudes toward reading. Our neediest students are also our most under-represented in terms of the books that are available to them. Our project would have these students create their own books and digital stories drawing on their cultural experience and sharing their stories with others. (Email communication, October, 2012).

The notion of identity texts (Cummins and Early, 2011) focuses on linking identity affirmation and literacy engagement. Students invest their identities in the creation of these “texts,” which can be written, spoken, signed, visual, musical, dramatic, or combinations in multimodal form. Through identity texts, students’ identities, cultures, languages, and past and present experiences are “reflected back in a positive light.” When students share identity texts with multiple audiences (peers, teachers, parents, grandparents, sister classes, the media, etc.) they are likely to receive positive feedback and affirmation of self in interaction with these audiences. In this classroom, the use of digital technologies, as well as the use of multimodal drama pedagogy, acted as an amplifier to enhance the process of identity text production.

As mentioned in the above email, the teachers were particularly interested in the creation of multilingual identity texts with their students, not only because the home languages of most of their students were not reflected in the bilingual or multilingual books available to them, but also because they thought that their students’ academic engagement and interest in literacy would increase if they could bring their knowledge of, and pride in, their cultures, identities and languages into their mainstream classrooms through the creation of multilingual texts.

The collaboration between these two Grade 3 teachers and the university-based researchers sought to create instructional approaches that would support students’ academic engagement in general, and literacy engagement in particular. Many different projects took place during the academic year, based on the curriculum expectations articulated by the provincial Ministry of Education, the two teachers’ specific questions, and projects that connected with students’ lives and interests, opening up the pedagogical space to include students’ home languages and cultural knowledge. Students were encouraged to write in their home languages, as well as in English (with the help of the school translator, their parents and their proficient peers). Our goal was to observe and document the literacy practices that emerged when the learning space was opened up to other languages in addition to English and when digital technology tools and drama pedagogy provided incentives and support for students to engage with multimodal forms of literacy. For the purposes of this paper, we will describe one of these projects, in which we explored how a descriptive writing unit could be implemented in a way that connected with students’ lives and enabled them to use their home language(s) in order to increase their engagement in learning and interest in literacy.

The claims to knowledge afforded by collaborative pedagogical inquiry rest in the documentation of teaching/learning interactions and their outcomes, which are brought about by the instructional initiatives undertaken. These claims are obviously not generalizable beyond the specific classroom contexts in which the initiatives were implemented and observed. However, the documentation of what happened in these pedagogical interactions constitutes phenomena that require explanation and are capable of refuting theoretical hypotheses. For example, the claim that students’ home languages cannot feasibly be mobilized for instructional purposes has been refuted by numerous examples deriving from this type of research (e.g., Chow and Cummins, 2003; Lotherington, 2011). The implications for policy can be summarized succinctly in the phrase *Actuality implies Possibility* – if a particular instructional initiative has been successfully implemented, then it *can* be implemented. Thus, our goal in the present study was to add to the documentation regarding the feasibility of undertaking instructional initiatives that position students’ home languages as cognitive and instructional resources.

## THEORETICAL FRAMEWORK

A number of theoretical lenses informed this work. Multiliteracies approach, initially proposed by the New London Group (1996) and elaborated subsequently by numerous researchers (e.g., Cope and Kalantzis, 2000, 2009; Hull and Schultz, 2001, 2002; Pahl and Rowsell, 2005; Anstey and Bull, 2006; Alexander, 2008; Gee, 2008; Jewitt, 2008; Mills, 2010; Lotherington, 2011; Yaman Ntelioglou, 2011; Heydon, 2012; Leander and Boldt, 2012; Hibbert, 2013) with its focus on multimodality, stresses the need for schools in the 21st century to focus on a broader range of literacies than simply traditional reading and writing skills, distinguishing itself from mainstream language and literacy theories by drawing attention to multiple modes of meaning making and communication (e.g., audio, visual, linguistic, spatial, performative) and how they can help students optimize their language and literacy learning. It also responds to the increasing cultural and linguistic diversity, paying attention to the importance of multilingualism and L1 use in the classroom.

Drawing on sociocultural and poststructural theories of identity and the notions of identity positioning (Toohey et al., 2007) and identity investment (Norton, 2000) is also important for our work, since, as Toohey et al. (2007) argue, “the formation and negotiation of identity positions represent an important dimension of classroom practices that contributes critically to students’ evolving relationship with school communities and their investment in learning English” (627). Based on the poststructural notions of identities as hybrid, multiple and dynamic, and the notion of identity positioning, classroom practices that draw on students’ funds of knowledge and linguistic and cultural capital help students to develop a positive sense of who they are and how they relate to their teachers, classmates and to the outside world. Literacy Engagement pedagogical framework and identity texts pedagogical practice, which we describe below, complement these notions of identity positioning, identity investment and literacy learning.

Literacy Engagement pedagogical framework (Cummins and Early, 2011) posits that literacy engagement is a major determinant of literacy achievement. This proposition is well-established empirically (e.g., Organisation for Economic Cooperation and Development [OECD], 2010) but has rarely been explicitly articulated in school improvement policies. The framework also highlights the importance of literacy engagement for (a) scaffolding meaning, (b) connecting to students’ lives, (c) affirming student identities, and (d) extending students’ awareness and command of academic language across the curriculum. There is general consensus among researchers, educators, and policy-makers about the importance of scaffolding meaning, connecting to students’ lives (e.g., by activating and building background knowledge) and extending language. This is illustrated by the fact that all three constructs are repeatedly invoked by the authors who were invited to contribute to the synthesis of research on ELLs published by the California Department of Education [CDE], (2010). However, the role of identity affirmation has not been generally acknowledged by policy-makers and many researchers. Thus, the Literacy Engagement framework differs from other school improvement tools insofar as it is focused on school improvement in schools serving multilingual students and highlights the role of both literacy engagement and identity affirmation as central components of effective instruction. The Literacy Engagement framework was used in the project as a starting point for discussion, among educators and researchers of the research evidence regarding effective pedagogical practice.

Linked to this pedagogical framework is the pedagogical practice of the creation of identity texts, described in a previous section. The basic claim underlying the concept of identity text is that students will engage actively with literacy only to the extent that such engagement is identity-affirming. In this regard, creative writing and other forms of cultural production (e.g., art, drama, computer animation) assume particular importance as an expression of identity, a projection of identity into new social spheres, and a re-creation of identity as a result of feedback from and dialog with multiple audiences. This re-creation of identity through the production of identity texts assumes particular importance in the case of students from social groups whose languages, cultures, religions, and institutions have been devalued, often for generations, in the wider society.

Finally, in developing our pedagogical initiatives, we took account of the need to acknowledge explicitly the multilingual and plurilingual realities of students’ linguistic repertories. A distinction between multilingualism and plurilingualism has been made by the Council of Europe (CECR) and scholars in North America such as Danièle Moore (Moore, 2006; Moore and Castellotti, 2008; Moore and Gajo, 2009), Heather Lotherington (Lotherington, 2013), and Enrica Piccardo (Piccardo, 2013). Plurilingualism refers to the dynamically integrated and intersecting nature of bi/plurilingual individuals’ linguistic repertoires, which include unevenly developed competencies in a variety of languages, dialects, and registers. Multilingualism, by contrast, in the Council of Europe’s framework refers to the presence of several languages in a given geographical area or social context, regardless of those who speak them (Coste et al., 2009; Beacco et al., 2010; Cenoz and Gorter, 2013; Piccardo, 2013). Moore and Gajo (2009) explain that multilingualism is “the study of societal contact” and that plurilingualism is “the study of individuals’ repertoires and agency in several languages” (p. 138). Citing the English version of the CECR, Moore and Gajo state that “plurilingual and pluricultural competence refers to the ability to use languages for the purposes of communication and to take part in intercultural interaction, where a person, viewed as a social agent has proficiency, of varying degrees, in several languages and experience of several cultures. This is not seen as the superposition or juxtaposition of distinct competences, but rather as the existence of a complex or even composite competence on which the user may draw” (CECR, English version, 2001, p. 168 as cited in Moore and Gajo, 2009). This nuanced understanding of plurilingual speakers as social actors developing a repertoire of multiple languages, and rarely equally or entirely fluent in all of their languages, was important to our study. In the context of our project, any attempt to connect instruction to students’ lives must take account of the fact that students speak multiple languages and have varying degrees of competence in them.

## RESULTS AND DISCUSSION: DESCRIPTIVE WRITING – MULTILINGUAL, MULTIGENERATIONAL DESCRIPTIONS OF FAVORITE PLACES IN SCHOOL

In this paper we describe only one of the multilingual projects in which students engaged. Our purpose is to illustrate the kinds of academic work that students with very limited English are capable of producing when teachers teach through a multilingual lens that acknowledges (1) the dynamically interconnected nature of their multiple languages and/or dialects and their relationships with each other (Piccardo, 2013); (2) that students’ competencies in these multiple languages can be unevenly developed; and 3) that not only multilingual students but all students, including those who are monolingual, benefit from a multilingual pedagogy by increasing their “Language Awareness,” that “has students attend systematically to language diversity and compare the patterns of their own languages as well as those of their classmates, communities, and the media” (Dagenais, 2005, n. page); and (4) that students develop metalinguistic awareness in these cross-linguistic learning environments (Duibhir and Cummins, 2012; Naqvi et al., 2014).

Descriptive writing was a curriculum expectation for Grade 3 students, and the two teacher/researchers found that this was a challenging task for the students in this class, who had a range of literacy levels from emerging to grade-level, for a number of reasons: most students had trouble moving beyond simple physical descriptions (e.g., big, green, wooden) to richer sensory and emotional analysis. Because many of ELL students were in the initial stages of reading or pre-reading and writing in English (and some with their other languages as well), their descriptions were further limited by gaps in vocabulary. Finally, some students were at such a beginning level of literacy awareness and practice, like some of the recently arrived Roma students, that engaging them in writing itself was the goal. In order to make their descriptive writing richer and more meaningful, we searched for a theme that would help them relate to and personalize their writing. We decided that the theme of “their favorite places within the school” could nurture a more descriptive, sensory, and emotional piece of writing. We also wanted them to experiment with multiple forms of text and multiple languages rather than being confined to traditional print-based text in English.

Each student first brainstormed about their favorite place and made a drawing of it. Then, each student took pictures of this favorite place using iPads. Next, they wrote a sensory and emotional description of what this special place meant for them. We recognized early on that students would become more engaged in the project if their parents and community were also involved. Therefore, we invited the parents and extended family members to be part of the project. For homework, the students interviewed their parents or an older family member about their own experiences in Grade 3 (or elementary school), and about their own favorite place or activity in the school they attended. Students took notes during the interview and brought these notes back to school. We invited the students to write both their own stories and their parents’ stories in other languages, if they wished, in addition to English.

As Toohey et al. (2007) suggest, “through participation in the social practices of the classroom, children develop a sense of the order of the academic world and their place within it, their status relative to teachers and peers, the nature of the tasks they face, and the relative legitimacy ascribed to their cultural and linguistic resources. For young second language learners, these broad lessons crucially influence investment in, access to, and acquisition of English” (626). The multilingual and multimodal practices in the classroom changed the power relations in the classroom and allowed the students access to identity positions of expertise, increasing their literacy investment, literacy engagement and learning. At the beginning of this project, the two teachers were worried because many of the newcomer ELL students, especially the Roma students, had developed “learned helplessness.” When they were asked to read or write any text, their immediate response was “Miss, I don’ t know how to read/write.” Both the multimodal practice and the multilingual practice changed this dynamic. For example, from the outset of the project, whenever we introduced the students to any technology, we decided to first teach the use of the technology (use of iPad, computer applications, etc.) to these ELL students so that they could become the experts, and later could teach their classmates, accessing their identity positions of expertise. The multimodal nature of the texts they created using digital media allowed them to express themselves in ways not limited to the linguistic mode, but multimodally using gestures, visuals, demonstrations etc. in addition to the linguistic mode. These multimodal affordances especially helped certain students who normally felt embarrassed about their lack of spoken language fluency.

**FIGURE 1:**
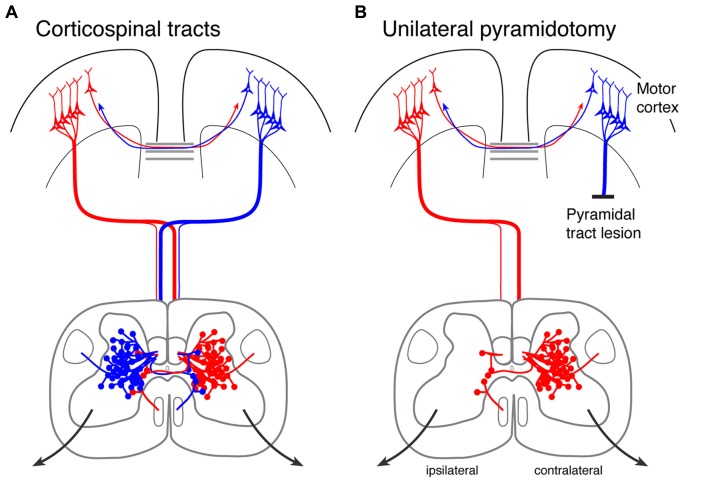
**Jose’s narrative**.

The multilingual focus also allowed the student access to identity positions of expertise. **Figure 1** shows Jose [pseudonym]’ s narrative, which is written both in English and Spanish. Jose was born in Canada, yet he was a fluent Spanish speaker since Spanish was regularly spoken at home. However, he had never been schooled in this language and so could not read or write in Spanish. On the other hand, his teacher, Jennifer, had studied Spanish as a foreign language and could read and write in that language, yet she never had the opportunity to become immersed in the language and develop a natural fluency. The picture of the two of them working on this project was intriguing: a standing student dictating his narrative to the seated teacher, who was trying to write it all down. There was a lively bilingual conversation between the two, since they were collaboratively deciding on certain vocabulary, expressions, and sentence structures that would best describe Jose’ s story. Jose’ s body language alone expressed the deep engagement he was experiencing in this literacy activity. He was in charge, directing the narration of his own story, and the teacher was the facilitator, working alongside her student to co-construct the text. This reversal of the traditional classroom dynamic (in which, generally, the teacher dictates to the student) resulted in the student having at least equal say in what the text was going to be about and how it was going to be told, which organically and inevitably shifted the power relations in the classroom. This kind of collaborative practice engaged the student by acknowledging his bilingual skills and maintaining his ownership of the narrative. This identity position of expertise, in turn, resulted in greater agency and a deeper level of investment as observed by the teachers and researchers.

**FIGURE 2:**
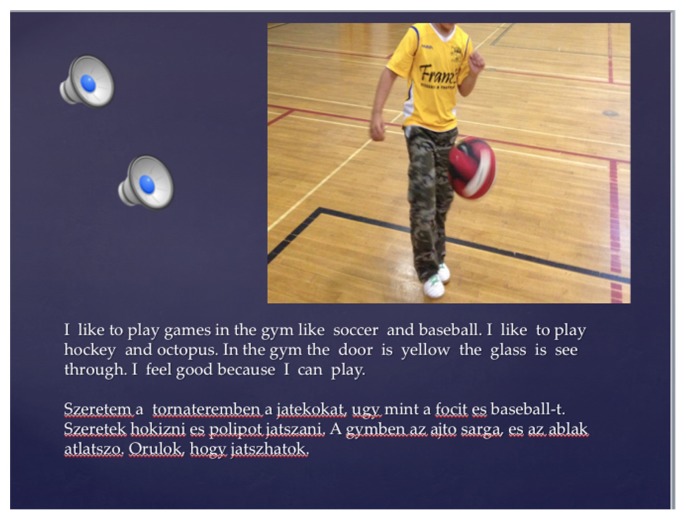
**Pali’s PowerPoint slide**.

Pali [pseudonym] was a Roma student and English was quite new to him. Therefore, Pali, like the other Roma students in his class, chose to write his narrative (**Figure 2**) initially in Hungar- ian. (It is important to note here that Pali wrote in Hungarian as opposed to Romani because he had been schooled in Hungarian for a year before he arrived in Canada. Most of the Roma students in this class spoke “street Hungarian” to get by; like Pali, some had also been schooled in Hungarian.) Roma students then found ways to express themselves either in shorter sentences in English, or in a direct translation from Hungarian with the help of a school-based translator^7^. Using different digital technologies such as PowerPoint, iMovie, iPhoto, and iPads, students were also able to record their own voices reading these multilingual stories. They also added images, songs, sounds and other modes of representation. Some of these narratives were also turned into dramatic performances. Drama practice was a particularly important aspect of the revision process in the student’ s writing of their individual narratives because, as they were trying to embody the narrative that was represented on the page, they could test the print representation against what they meant to convey. This kind of dialogic feedback afforded through embodied multimodality (Yaman Ntelioglou, 2011, forthcoming) helped to immediately see what was working and what was not working with their writing.

Even students who did not have strong fluency in their home languages, because they were born in Canada and/or had been schooled here from the age of four, and who did not regularly speak their family’ s first language at home, responded positively to the invitation to use their home languages.

**FIGURE 3:**
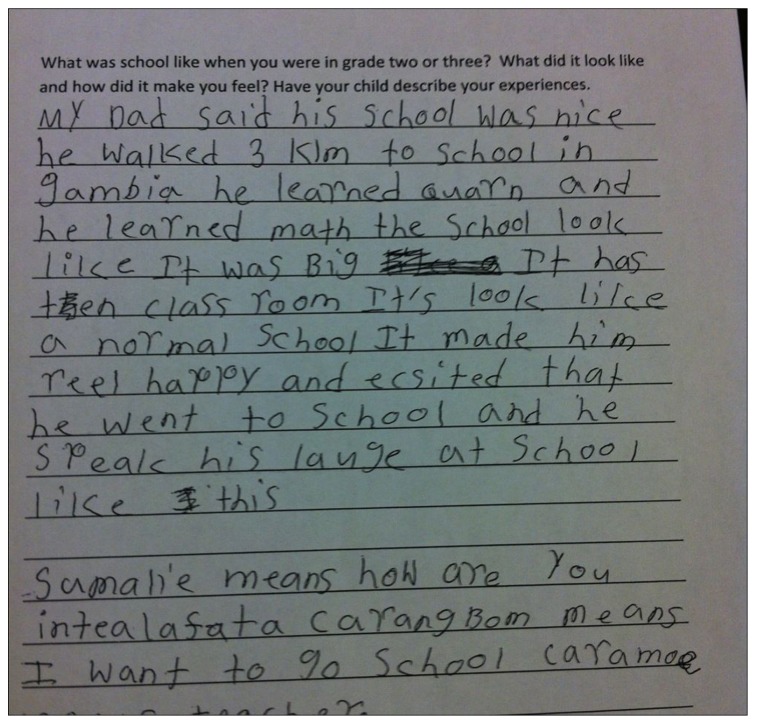
**Fatu’s first draft**.

**FIGURE 4:**
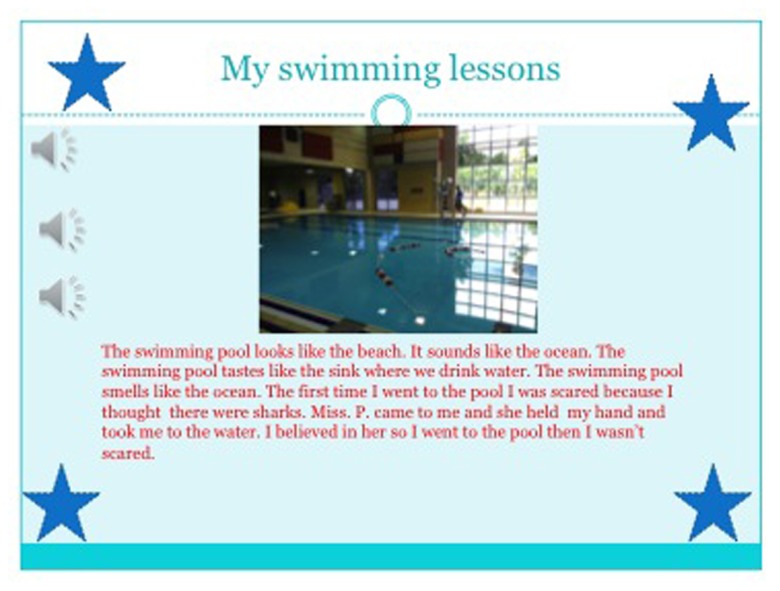
**A slide from Fatu’s PowerPoint presentation**.

**FIGURE 5:**
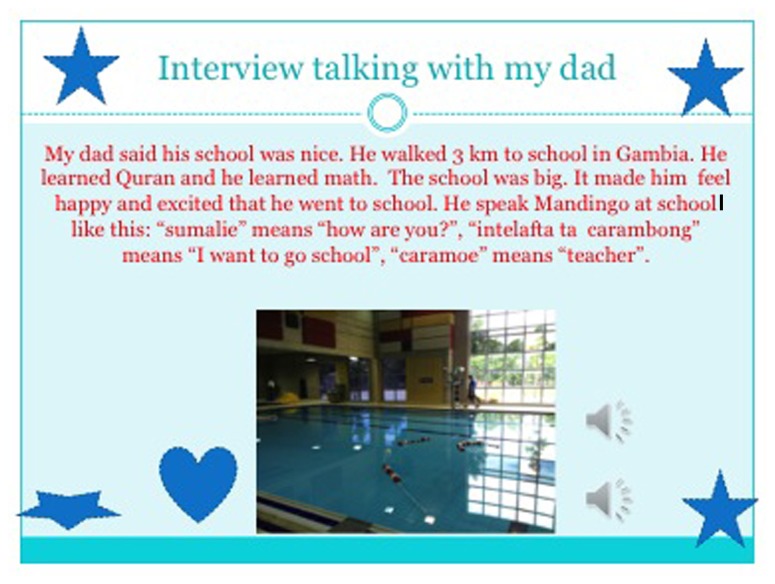
**Another slide from Fatu’s PowerPoint presentation**.

Because students were invited to write in multiple languages, some students like Fatu [pseudonym] -who said she sometimes understood her home language, Mandingo, but did not speak it - chose to include some Mandingo words in her writing. Fatu’ s parents are from Gambia and Fatu was born in Canada. At home the parents sometimes spoke Mandingo, but they resorted to English mostly when they spoke with Fatu and her siblings. As seen in Fatu’ s first draft (**Figure 3**), as well as a subsequent draft in PowerPoint (**Figure 5**), based on the interview that she did with her father, she wrote about how he walked three kilometers to school in Gambia and that he learned the Quran. She adds, “Sumalie” which she explains means “how are you;” “intelafta ta carambong” means “I want to go to school;” and “caramoe” means “teacher.” This curiosity about her home language carried on past the time of the assignment. For example, one day she came to us and excitedly asked if we could videotape her with the iPad, because she now knew how to count in Mandingo. This is another example of how affirming students’ multilingual and multicultural *funds* of knowledge (Gonzalez, 1995) can nurture their identities and their investment in learning, not only in their L2, but also in their L1, and in turn, foster learner autonomy (Benson, 2006; Jiménez Raya, 2009). Benson (2006) draws attention to the social dimensions of learner autonomy, and in reference to Toohey and Norton’ s (2003) conception of identity investment and agency, state that “agency can perhaps be viewed as a point of origin for the development of autonomy, while identity might be viewed as one of its more important outcomes” (30). As this project proceeded, Fatu became a very prolific writer and story-teller. According to her teachers, “she developed from a learner who showed initial enthusiasm for school work, but less carry-through, to a learner who was more engaged, autonomous and more able to see the work through to completion” (Teacher focus interview, February 2012). She completed the writing project and went beyond the basic requirements, adding a narrative in both of her languages as well as a song, making her narratives more multimodal as well as more representative of her identities. In the PowerPoint slides, as seen below in **Figure 4**, the two audio buttons on each slide were linked with the audio segments she recorded in English and Mandingo, even though as explained above, her proficiency in Mandingo was not advanced. It is also interesting to note that her Mandingo recordings are done in a much lower volume, which may also reflect her relative lack of confidence, but she persisted nevertheless. In the interview with Fatu, she explained that she wanted to show her audience who she is by including both of her languages, as well as a song (audio button 3), in her PowerPoint because she loves singing. She explained that for the moment she can only sing in English, but her goal is to also learn to sing in Mandingo. For her PowerPoint, when she was recording herself in Mandingo, and encountered words like “ocean” and “pool,” for which she did not have equivalent Mandingo words, she altered English words by adding the final suffix –o, to make them sound more like Mandingo words. Her elder sibling, in an informal conversation, explained that the final vowel in most Mandingo words is –o. This simple translanguaging example and Fatu’ s explanations of this cognitive process in the interview saying, “in Mandingo, most words for things end in –o” shows her metalinguistic awareness.

Mixing of languages (if conceptualized as “code-switching”) can be seen as an error, a “dangerous flaw,” when approached from a traditional bilingual perspective that assumes that the two languages of bilinguals are two separate monolingual codes. However, from the perspective of plurilingualism (Moore and Gajo, 2009)/multilingualism (García, 2009), Fatu’ s mixing of English word (pool) with the Mandingo suffix “-o” is a valuable translanguaging practice that illustrates that bilinguals have one linguistic repertoire from which they select different features strategically, to communicate more effectively. 

Fatu’ s story was also one of the narratives that was turned into a dramatic performance. Students worked in groups and decided how they would like their individual narratives to be performed. They had options regarding which role to take on, which props they were going to use, and how they were going to bring the story alive. In the performance of her story, Fatu chose the role of storyteller, while three other classmates acted it out. Sequence one depicted her favorite place in school, and sequence two depicted her father’ s story. As a result of transferring the written text back and forth into the embodied, students were asked to consider “the content and context of the statements, and provided a forum that allowed for communication, restating and subsequent interaction” (Booth, 1991, p. 95). Students became aware of their own weaknesses and problems in writing by reading each others’ writing and working collaboratively. The multiple voices of each of the four students (one Mandingo, one Spanish and two Tibetan speakers) informed the embodied collective creation. Fatu’ s group decided that since they were coming from multiple linguistic backgrounds, it would be a good idea to begin the performance by saying the title of the story in their multiple languages, and ending the performance by saying goodbye, using the words and gestures of their respective home languages and cultures.

In the classroom, having the opportunity to use their multiple languages, through multimodal texts, students had the opportunity to choose their multiple linguistic repertoires, their medium of choice(s) to express their meanings. Having these multiple options and choices allowed the students to make their texts/narratives their own, fostering learner autonomy, identity investment, and literacy engagement. Even though translanguaging practices were not explicitly taught in this class, because students were invited to use their multiple linguistic repertoires, some students naturally used translanguaging practices, drawing on all of their linguistic resources “to maximize understanding, (self-expression), and achievement. Thus, both languages (were) used in a dynamic and functionally integrated manner” (Lewis et al., 2012, p. 655), illustrating that the two or more languages of bilinguals or plurilinguals do not function as two or more separate monolinguingual codes; rather they exist as a holistic and interactive linguistic repertoire (García, 2009; Lewis et al., 2012).

## CONCLUSION

As noted in our introductory section, there is a void with respect to language policy in schools, school boards, and Ministries of Education across Canada. This neglect is highly problematic because, in the absence of any coherent articulated policies, the “default option” will be to ignore students’ languages, cultures, and background knowledge within schools and classrooms. Schools then become “English-only zones” (or “French-only zones” in Quebec, as well as in French-immersion programs in various provinces, Taylor, 2010; and Franco-Ontarian schooling, Russette and Taylor, in press), which reinforces the societal pattern of power relations whereby the cultural capital or funds of knowledge of dominant group communities are valued considerably more than the cultural capital of the many other communities that make up the Canadian social landscape.

However, educators have the power to exercise agency in relation to the ways in which they negotiate identities with their students (Cummins, 2001). As our case study documents, enlightened language policies can be implemented by individual teachers in their own classrooms. Furthermore, these policies are considerably more evidence-based than English-only zone policies insofar as they (a) promote students’ literacy engagement, (b) scaffold comprehension and production of academic language, (c) connect with students’ lives and activate their background knowledge, (d) affirm students’ identities as linguistically talented and intellectually accomplished, and (e) extend and deepen students’ awareness of academic language. When teachers open up the instructional space for multilingual and multimodal forms of pedagogy, languages other than English or French are legitimized in the classroom and students’ home languages and community connections become resources for learning.

## Conflict of Interest Statement

The authors declare that the research was conducted in the absence of any commercial or financial relationships that could be construed as a potential conflict of interest.

## Footnotes

1 http://www.elodil.com/

2 http://www.thornwoodps.ca/dual/index.htm

3 http://www.multiliteracies.ca

4 http://www.rahatnaqvi.ca

5 http://www.duallanguageproject.com/

6 http://www.scribjab.com

7 In 2009, during the second year of the influx of Roma students, the principal hired a part-time Hungarian translator. A Hungarian, rather than Romani, translator was hired because there were no Romani translators available and, according to the Hungarian translator, Hungarian Romani is a particular type of Romani, different from the type of Romani that might be spoken in other countries. Since all students had some fluency in street Hungarian, and some had been schooled in Hungarian, the school was advised to hire a Hungarian translator.
